# Stroke patients have lower blood levels of nutrients that are relevant for recovery: a systematic review and meta-analysis

**DOI:** 10.3389/fstro.2023.1274555

**Published:** 2023-12-13

**Authors:** Laus M. Broersen, Sonia Guida, Aysun Cetinyurek-Yavuz, Nick van Wijk, Ardy van Helvoort, Adina T. Michael-Titus, Mirian Lansink

**Affiliations:** ^1^Danone Nutricia Research, Utrecht, Netherlands; ^2^Department of Health Evidence, Radboud University Medical Center, Nijmegen, Netherlands; ^3^Department of Respiratory Medicine, NUTRIM - School of Nutrition and Translational Research in Metabolism, Maastricht University Medical Center+, Maastricht, Netherlands; ^4^Centre for Neuroscience, Surgery and Trauma, Barts and The London School of Medicine and Dentistry, The Blizard Institute, Queen Mary University of London, London, United Kingdom

**Keywords:** stroke, malnutrition, neurorehabilitation, nutrient deficiency, blood levels

## Abstract

**Background:**

Malnutrition is common after stroke. Stroke patients often have a suboptimal energy intake, body weight and inadequate blood nutrient levels. Nutrient insufficiencies may not be detected, but their recognition is essential to provide adequate nutritional support after a stroke. This comprehensive summary of the literature is a collection of data on blood levels of a broad selection of nutrients involved in restoring cerebral blood flow and functional brain connectivity in stroke patients compared to controls.

**Methods:**

Embase and MEDLINE were searched for studies published in English in the period 1980–2022. Studies including adult stroke subjects and controls whose blood samples were analyzed for docosahexaenoic acid (DHA), eicosapentaenoic acid (EPA), uridine, choline, folate, vitamin B6, vitamin B12, vitamin C, vitamin E, selenium, coenzyme Q10 (CoQ10), carnitine, arginine, or taurine were selected. If there were >3 reports (*k*) per nutrient, results were generated with an unadjusted and age-adjusted random-effects meta-analysis model. Risk of bias was evaluated for relevant domains from the ROBINS-I tool and with Egger's test.

**Results:**

One hundred five reports on blood nutrient levels were extracted from 56 eligible studies. Overall, meta-analyses showed lower blood levels of most nutrients in stroke patients compared to controls. The number of reports and the statistical significance for the unadjusted data were: folate (*k* = 27; *p* = 0.005), vitamin B12 (*k* = 23; *p* = 0.002), vitamin E (*k* = 11; *p* = 0.013), DHA (*k* = 7, *p* = 0.015), EPA (*k* = 7; *p* = 0.004), vitamin C (*k* = 6; *p* = 0.020), and selenium (*k* = 6; *p* = 0.018). No significant decreases were observed for vitamin B6 (*k* = 6; *p* = 0.52) and arginine (*k* = 4; *p* = 0.93). For other selected nutrients, there were insufficient reports to perform a meta-analysis. Available reports pointed toward lower (CoQ10, choline; *k* = 2), higher (taurine; *k* = 2), or unchanged (carnitine, uridine; *k* = 1) blood levels after stroke. In general, risk of bias was low.

**Conclusion:**

Our findings indicate that nutrient insufficiencies occur for many nutrients that are involved in repair processes after stroke. The low blood levels of folate, vitamin B12, EPA, DHA, vitamin C, vitamin E, selenium, and possibly CoQ10 and choline, highlight the presence of a suboptimal nutritional status after stroke. The inclusion of targeted nutritional interventions to further support recovery should receive consideration in the multidisciplinary context of stroke rehabilitation.

## 1 Introduction

Malnutrition after stroke is common (Burgos et al., 2018) and occurs across the continuum of stroke care, from the hyperacute to the chronic phase (Huppertz et al., 2021). Malnourishment in stroke patients may not be limited to protein-energy malnutrition and can also include insufficiencies or deficiencies in specific micronutrients and fatty acids, reflected by their lower blood levels (Cherubini et al., 2000; Han et al., 2002; Ikeya et al., 2013). Reduced levels of circulating nutrients can be related to a reduced dietary intake, be a consequence of previous or current lifestyle, be due to medication influencing the absorption and excretion of nutrients, or be due to condition-specific metabolic changes that alter nutrient requirements (Lieber et al., 2018; Sabbouh and Torbey, 2018; Chen et al., 2019; Wakeman and Archer, 2020).

Impaired nutritional status after stroke has consistently been associated with poor outcome and reduced functional recovery (Gariballa et al., 1998; Martineau et al., 2005; Yoo et al., 2008; Gomes et al., 2016). Apart from protein-energy undernutrition, this may be due to insufficiencies in specific nutrients that are relevant for clinical outcome after this central nervous system injury. The acute response to ischemia is characterized by cell death, the formation of free radicals and reactive oxygen species, and an inflammatory response. In the days and weeks following the ischemic event, adaptive brain plasticity mechanisms come into play, involving the release of different growth factors to support angiogenesis, gliogenesis, neurogenesis, neurite outgrowth, and synaptogenesis, that collectively contribute to the initiation of a (partial) recovery of function (Wieloch and Nikolich, 2006; Dalise et al., 2014). Physiological processes occurring after a stroke, which are part of the injury response and the emergence of repair mechanisms, depend on the presence of compounds derived from the body's nutrient reserves or from dietary intake. For instance, many nutrients are known for their antioxidant (Cheli and Baldi, 2011; Ruskovska et al., 2020), anti-inflammatory (Sanderson and Croft, 2005; Tyrovolas et al., 2018), or immune modulating properties (Suchner et al., 2000; Ruiz-Leon et al., 2019), all of which are relevant after stroke. In addition, nutrients act as cofactors in metabolic processes such as the one-carbon metabolism, where the presence of sufficient B-vitamins regulates homocysteine levels (Taylor et al., 2018). Furthermore, nutrients serve as precursors for signaling molecules and structural components, i.e., the building blocks for glial and neuronal cells, neurite extensions and synaptic connections, and new blood vessels that support their functioning. In order to adequately address the condition-specific nutritional needs of stroke patients and support an optimal rehabilitation, it is important to identify the most relevant nutrients which may be suboptimal.

Nutrient insufficiencies may not be detected, but their recognition is essential to provide adequate nutritional support to patients from the early phase post stroke. Therefore, we performed a systematic review and meta-analysis on reported blood levels of micronutrients, amino acids, and fatty acids shown to be involved in the restoration of cerebral blood flow and functional brain connectivity after stroke (Wurtman et al., 2010; van Wijk et al., 2014; Wiesmann et al., 2017). In particular, we investigated reported blood levels of docosahexaenoic acid (DHA), eicosapentaenoic acid (EPA), uridine, choline, folate, vitamin B6, vitamin B12, vitamin C, vitamin E, selenium, coenzyme Q10 (CoQ10), carnitine, arginine, and taurine in stroke cases compared to controls.

## 2 Materials and methods

### 2.1 Literature search

The systematic literature search was performed by a librarian specialist in two electronic databases, Embase and MEDLINE, to identify studies published in English in the period 1980–2022 in accordance with the PRISMA guidelines. Additional records were identified from recently published literature in 2023. The search strategy used to retrieve the relevant database records published between January 1980 and September 2022 was composed of a conceptual string with the following combination of terms or their analogs or synonyms: “cerebrovascular” AND “ischemic” AND “malnutrition” AND “DHA OR EPA OR uridine OR choline OR folate OR vitamin B6 OR vitamin B12 OR vitamin C OR vitamin E OR selenium OR taurine OR arginine OR CoQ10 OR carnitine” NOT “animal.” Non peer reviewed sources, such as conference abstracts, book chapters, and video-audio media were excluded. The full search strategy is available in the Supplementary Table S1. The review was not registered; no protocol was prepared for registration.

### 2.2 Eligibility criteria and study selection

Records were screened based on title and/or abstract and were excluded in case of reviews, case reports, duplicates and when the search criteria were not met. For example, studies that did not present data on any of the nutrients within the scope of this systematic review were excluded. The studies were assessed based on the full-text and excluded if the study population was <18 years old, or if the population included exclusively subjects with haemorrhagic stroke or transient ischemic attack, or it did not include a control group. The inclusion of a study in the meta-analysis required that the blood levels of the nutrients and the number of subjects assessed were available for the stroke and control group. Studies presenting data for more than one nutrient or for independent subgroups were treated independently and constituted the total number of reports. Study selection and data extraction was performed by three independent reviewers and the final dataset was discussed with the review team.

### 2.3 Outcomes

The blood levels of DHA, EPA, uridine, choline, folate, vitamin B6, vitamin B12, vitamin C, vitamin E, selenium, CoQ10, carnitine, arginine, and taurine in stroke cases and controls were extracted. Data on the type of stroke and timing after stroke were recorded. Timing was based on the definition of the stroke phases provided by the Stroke Recovery and Rehabilitation Roundtable Taskforce (Bernhardt et al., 2017), simplified to: acute (<7 days), subacute (between 7 days and 6 months), and chronic (6 months or more). Data on the type of controls were also extracted. Mean age and the number of subjects assessed were collected for both the stroke and control groups.

### 2.4 Risk of bias

Risk of bias (high, unclear, or low) was evaluated by two independent assessors for each study on the basis of 4 domains: selection, detection, attrition, and reporting bias. These were selected from the ROBINS-I tool (Sterne et al., 2016) as being most relevant for non-randomized observational studies. In short, each assessor rated the risk per domain as either high, unclear, or low. Then, all assessments were compared and sporadic disagreements were discussed between assessors to come to an agreement on the final score.

In addition, publication bias was assessed with Egger's test if possible (see Section 2.5).

### 2.5 Statistical analysis

Blood levels of the nutrients were expressed as mean ± standard deviation (SD). In the studies where the mean ± SD was available in subgroups rather than for the whole group of stroke patients or controls, the weighted mean and the pooled SD were calculated. When a study reported the mean value but the SD was missing the SD was estimated based on the standard error of the mean (SEM) or the figures provided in the study, if available. When a study reported median, first and third quartile, the formulas from Wan et al. (2014) were used to impute mean and SD. In case the median was available with the range of the data, the formulas from Hozo et al. (2005) were used to impute mean and SD.

To allow comparison across studies, the standardized mean difference (SMD) estimated by Hedges' *g*, was used to standardize the results of the studies to the same scale, which expresses the size of the effect in each study relative to the variability observed in that study.

In the meta-analyses where the number of reports was >3, the overall effect was estimated with a random-effect (RE) meta-analysis model with Hartung Knapp (HK) modification for unadjusted data. The analysis was replicated including age as a covariate if the number of reports was >3; age-adjustment was obtained by correcting for centralized age differences between groups. In case the mean age was not available and the median (range) was reported, or the mean age was available only in the stroke or the control group and these groups were age matched, the mean value of the age was imputed. The between-study variance was estimated using the *I*^2^ statistic, where an *I*^2^ > 95% indicates considerable amount of heterogeneity.

The publication bias was evaluated with Egger's test (regression test for funnel plot asymmetry) if the number of reports included in the meta-analysis was ≥10. The RE meta-analysis with HK modification and the Egger's test output was generated using Metafor package in R (Viechtbauer, 2010; R Core Team, 2022).

## 3 Results

**Figure 1:**
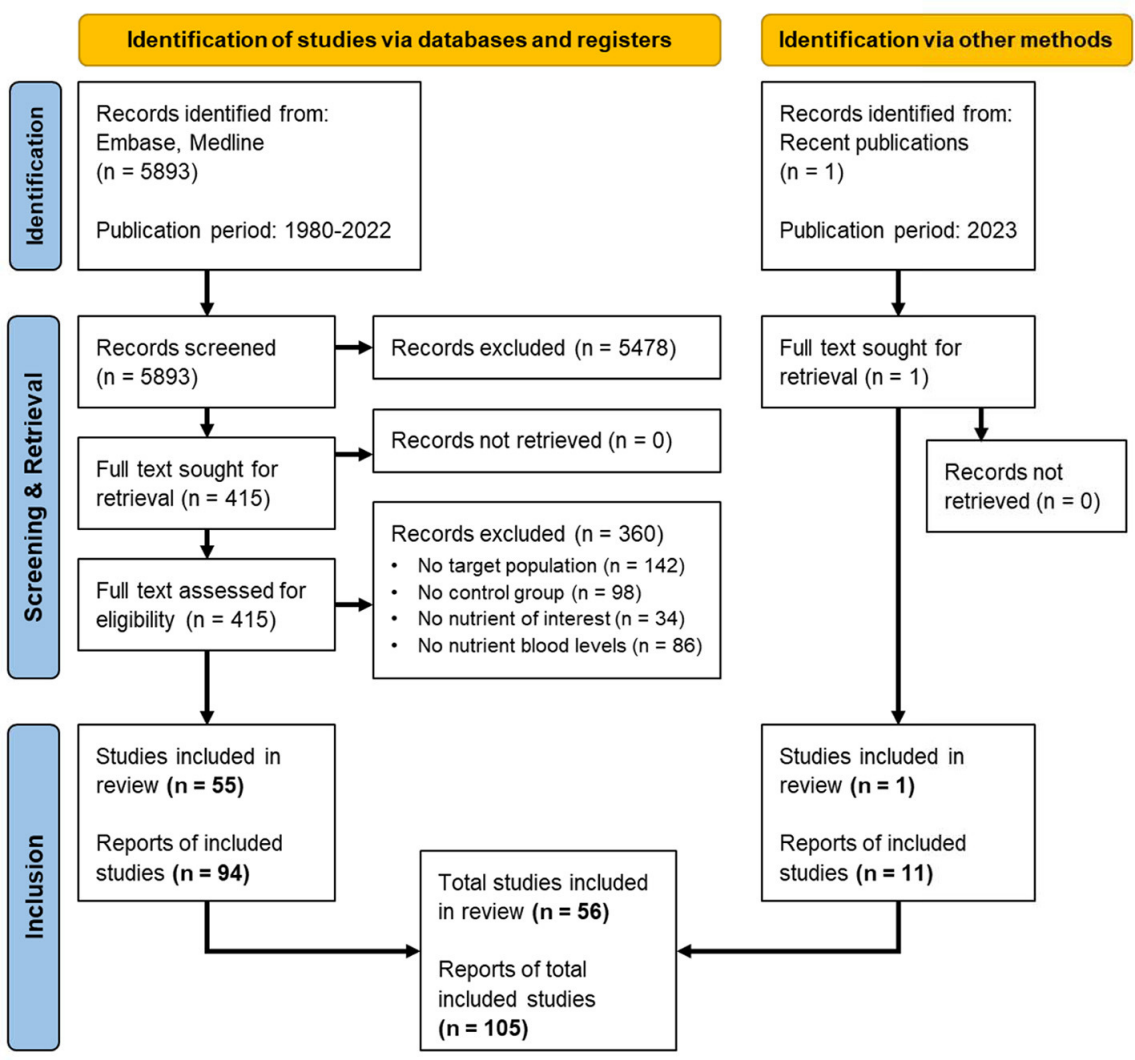
PRISMA flow diagram for the systematic review involving search of databases, registers and other sources, adapted from Page et al. (2021). Breakdown of the screening and retrieval, leading to the inclusion of 56 eligible studies and 105 reports for systematic review and meta-analysis.

The number of records identified from Embase and Medline was 5,893 for the publication period 1980–2022. A recent study (van Wijk et al., 2023) was identified as an additional record from recently published literature. Screening based on the eligibility criteria resulted in a total of 56 studies and 105 reports published in the period 1980–2023 (Figure 1).

### 3.1 Study characteristics

The number of stroke subjects included in the 105 reports was 5,725 and the mean age was 64.1 (range: 27.7–77.2) years. Overall, the main diagnosis was ischemic stroke and the assessment 165 of the nutrient blood levels occurred within the acute (*k* = 59), the subacute (*k* = 22), or the chronic (*k* = 11) 166 phase. For the remaining reports (*k* = 13), the moment of assessment was not indicated. Two studies with assessments overlapping phase borders, were assigned to the best fitting phase, i.e., 1–10 days (Han et al., 2002) to acute, and 21–42 weeks (Brattstrom et al., 1992) to chronic. The number of controls included in the reports was 16,171 and it primarily included relatively healthy subjects whose mean age was 60.6 (range: 27.0–77.8) years. The results of the 105 reports consisted of the following meta-analysis groups: folate (*k* = 27), vitamin B12 (*k* = 23), vitamin E (*k* = 11), DHA (*k* = 7), EPA (*k* = 7), vitamin B6 (*k* = 6), vitamin C (*k* = 6), selenium (*k* = 6), arginine (*k* = 4). Nutrients with three or fewer available reports, i.e., taurine (*k* = 2), CoQ10 (*k* = 2), choline (*k* = 2), carnitine (*k* = 1), and uridine (*k* = 1), were not included in the meta-analysis and were treated separately. A summary of the results is reported in Supplementary Table S2.

### 3.2 Risk of bias

**Figure 2:**
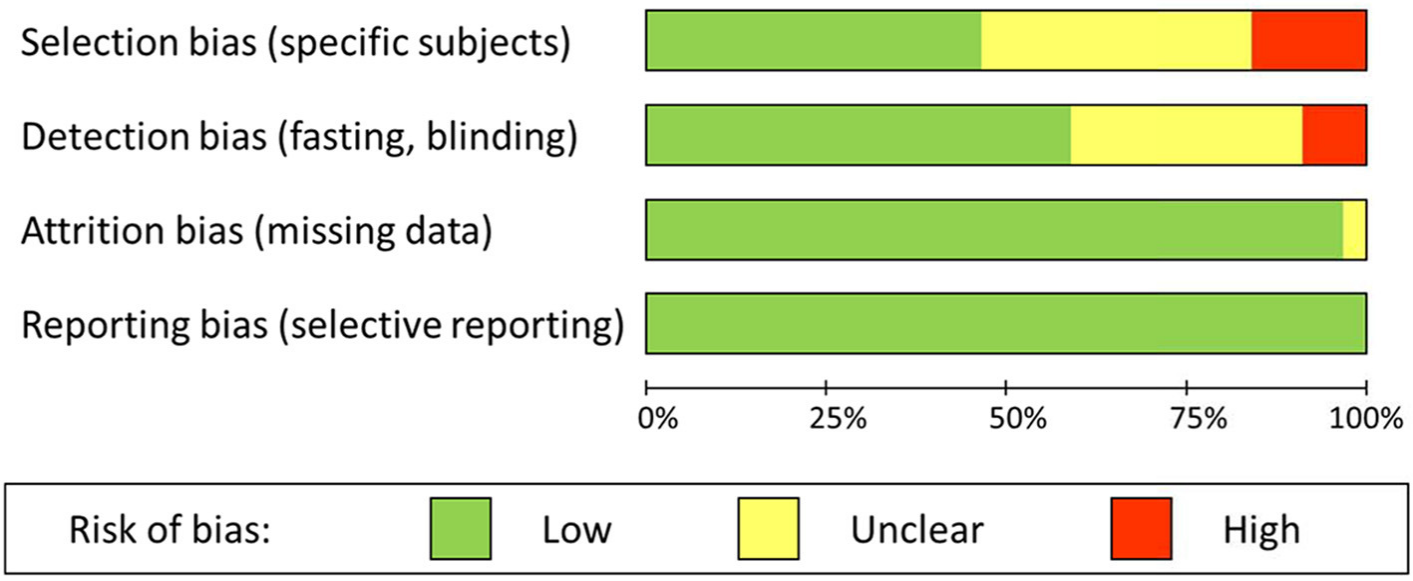
Summary of the risk of bias assessment results for the 56 included studies.

Risk of selection bias was present in 16%, and unclear in 38% of the studies. Selection bias was obvious where specific patients with a higher risk of malnutrition due to dysphagia or the use of specific medication were excluded. Similarly, the selection of relatively unhealthy control subjects, such as elderly patients without cerebrovascular diagnosis, was considered a source of bias. Risk of detection bias was present in 9%, and unclear in 32% of the studies. In most cases detection bias was due to a lack of fasting in one or both groups (stroke patients and controls) before blood sampling. In addition, unblinded handling or analysis of samples could have introduced bias. Risk of attrition bias and risk of reporting bias were very low to absent in the selected studies; there were hardly any missing data, and all outcomes were clearly reported. Overall results of the risk of bias assessment are summarized in Figure 2.

### 3.3 Folate

**Figure 3:**
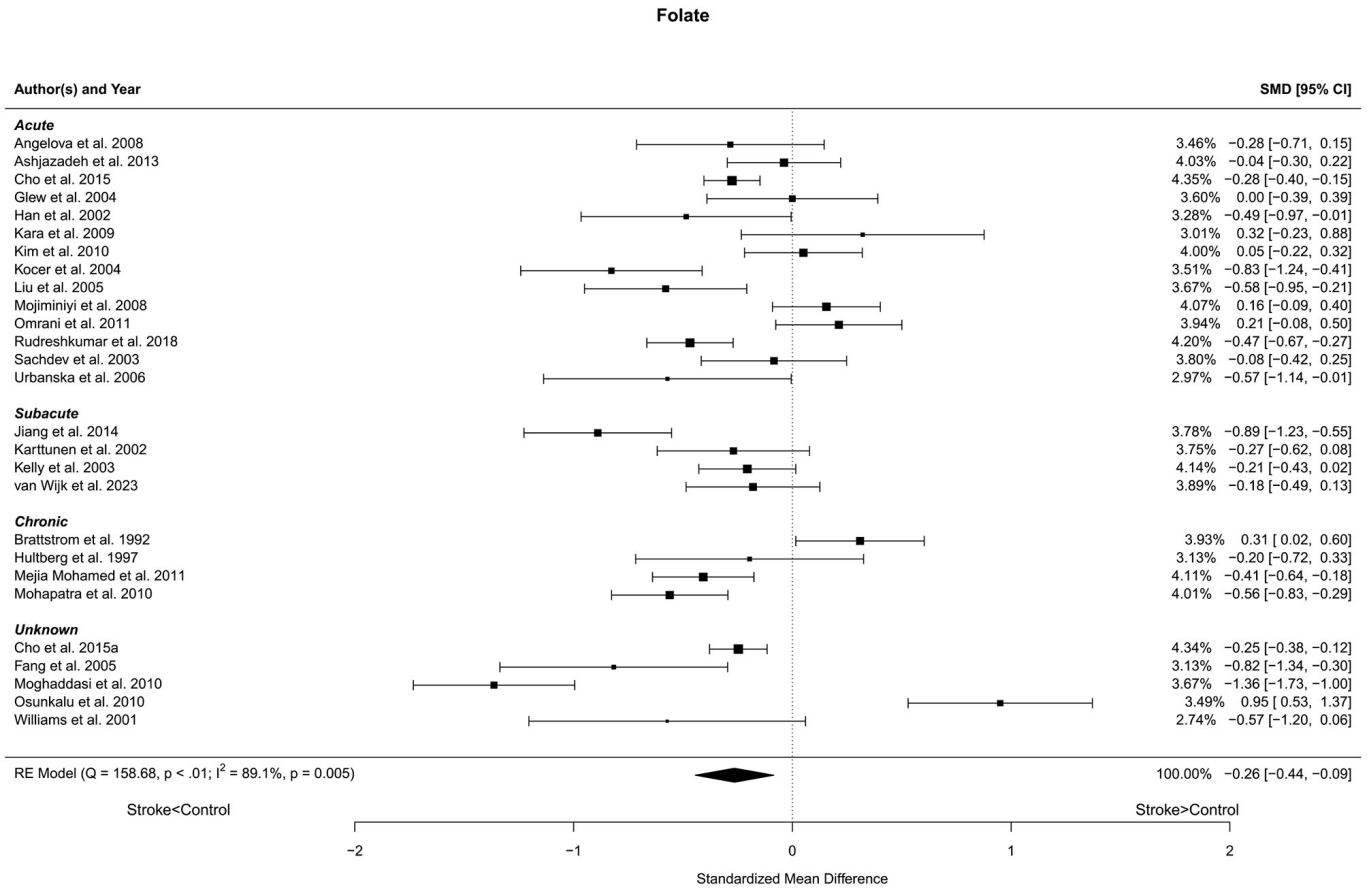
Comparison of blood folate levels in stroke patients and controls. Included reports are organized in the forest plot by phase (acute—subacute—chronic—unknown) to visualize possible effects of time after stroke. Relative weights (%) and standardized mean differences (plus confidence interval) for each report are indicated at the right. Characteristics and result of the RE meta-analysis are in the lower left corner.

Twenty-seven reports with a total of 3,279 stroke cases and 2,679 controls were included in the meta-analysis (Brattstrom et al., 1992; Hultberg et al., 1997; Williams et al., 2001; Han et al., 2002; Karttunen et al., 2002; Kelly et al., 2003; Sachdev et al., 2003; Glew et al., 2004; Kocer et al., 2004; Fang et al., 2005; Liu et al., 2005; Urbanska et al., 2006; Angelova et al., 2008; Mojiminiyi et al., 2008; Kara et al., 2009; Kim et al., 2010; Moghaddasi et al., 2010; Mohapatra and Sarangi, 2010; Osunkalu et al., 2010; Mejia Mohamed et al., 2011; Omrani et al., 2011; Ashjazadeh et al., 2013; Jiang et al., 2014; Cho et al., 2015a,b; Rudreshkumar et al., 2018; van Wijk et al., 2023). The mean number of subjects was 90.2 (range: 20–579) in the stroke groups and 99.2 (range: 20–427) in the control groups. The mean age was 60.8 (range: 27.7–72.7) years in the stroke cases and 58.6 (range: 27.0–71.7) years in the controls. Blood levels of folate were lower in stroke patients compared to controls for unadjusted [*t*_(26)_ = −3.03, *p* = 0.006] and age-adjusted [*t*_(25)_ = −2.97, *p* = 0.006] model (Figure 3). The Egger's test [*t*_(25)_ = −0.59, *p* = 0.56] indicated no evidence of publication bias (Supplementary Figure S1).

### 3.4 Vitamin B12

**Figure 4:**
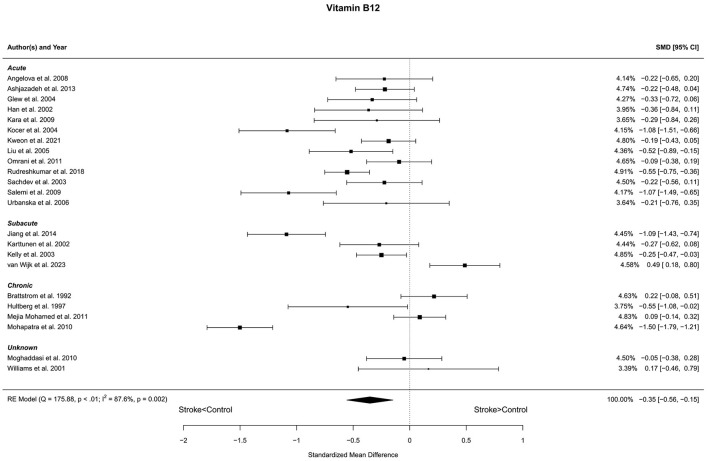
Comparison of blood vitamin B12 levels in stroke patients and controls. Included reports are organized in the forest plot by phase (acute—subacute—chronic—unknown) to visualize possible effects of time after stroke. Relative weights (%) and standardized mean differences (plus confidence interval) for each report are indicated at the right. Characteristics and result of the RE meta-analysis are in the lower left corner.

Twenty-three reports with a total of 2,075 stroke cases and 1,707 controls were included in the meta-analysis (Brattstrom et al., 1992; Hultberg et al., 1997; Williams et al., 2001; Han et al., 2002; Karttunen et al., 2002; Kelly et al., 2003; Sachdev et al., 2003; Glew et al., 2004; Kocer et al., 2004; Liu et al., 2005; Urbanska et al., 2006; Angelova et al., 2008; Kara et al., 2009; Salemi et al., 2009; Moghaddasi et al., 2010; Mohapatra and Sarangi, 2010; Mejia Mohamed et al., 2011; Omrani et al., 2011; Ashjazadeh et al., 2013; Jiang et al., 2014; Rudreshkumar et al., 2018; Kweon et al., 2021; van Wijk et al., 2023). The mean number of subjects was 90.2 (range: 20–199) in the stroke groups and 74.2 (range: 20–249) in the control groups. The mean age was 61.5 (range: 27.7–72.7) years for stroke cases and 59.7 (27.0–71.7) years for controls. Blood levels of vitamin B12 were lower in stroke patients compared to controls for unadjusted [*t*_(22)_ = −3.58, *p* = 0.002] and age-adjusted [*t*_(21)_ = −3.55, *p* = 0.002] model (Figure 4). The Egger's test [*t*_(21)_ = −0.27, *p* = 0.79] indicated no evidence of publication bias (Supplementary Figure S2).

### 3.5 Vitamin E

**Figure 5:**
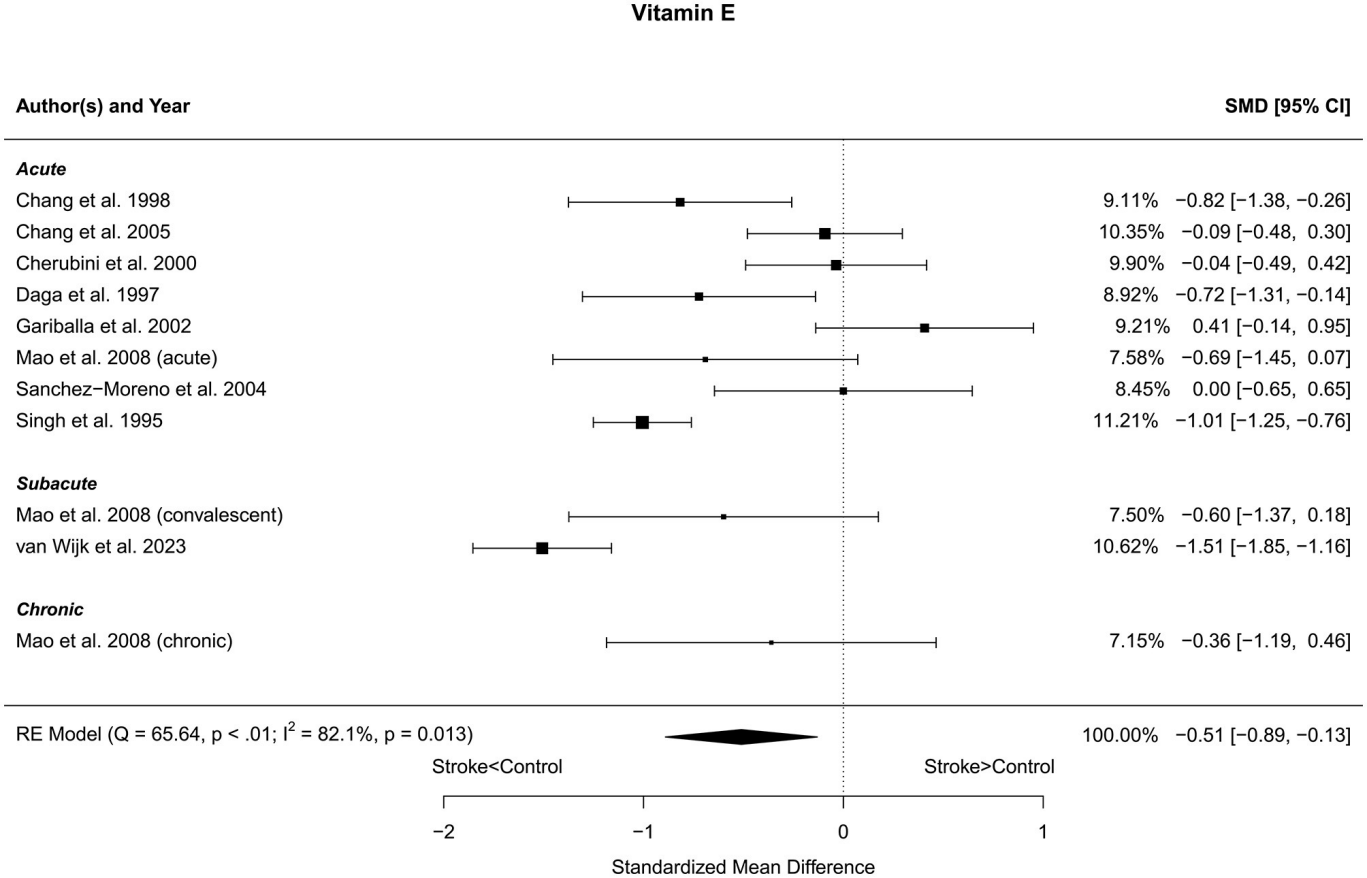
Comparison of blood vitamin E levels in stroke patients and controls. Included reports are organized in the forest plot by phase (acute—subacute—chronic) to visualize possible effects of time after stroke. Relative weights (%) and standardized mean differences (plus confidence interval) for each report are indicated at the right. Characteristics and result of the RE meta-analysis are in the lower left corner.

Eleven reports with a total of 457 stroke cases and 484 controls were included in the meta-analysis (Singh et al., 1995; Daga and Madhuchhanda, 1997; Chang et al., 1998, 2005; Cherubini et al., 2000; Gariballa et al., 2002; Sanchez-Moreno et al., 2004; Mao et al., 2008; van Wijk et al., 2023). Mao et al. (2008) reported three subgroups and one control group. Therefore, the control group was divided into three equal subgroups and using the same mean and SD for each control group. The mean number of subjects was 41.5 (range: 12–110) in the stroke groups and 53.8 (range: 20–202) in the control groups. The mean age was 65.9 (range: 52.5–77.2) years for stroke cases and 62.9 (range: 46.2–77.8) years for controls. The blood levels of vitamin E were lower in stroke patients compared to controls for the unadjusted [*t*_(10)_ = −3.00, *p* = 0.013] and for the age-adjusted [*t*_(8)_ = −2.56, *p* = 0.034] model (Figure 5). The Egger's test [*t*_(9)_ = 0.93, *p* = 0.38] indicated no evidence of publication bias (Supplementary Figure S3).

### 3.6 Docosahexaenoic acid

**Figure 6:**
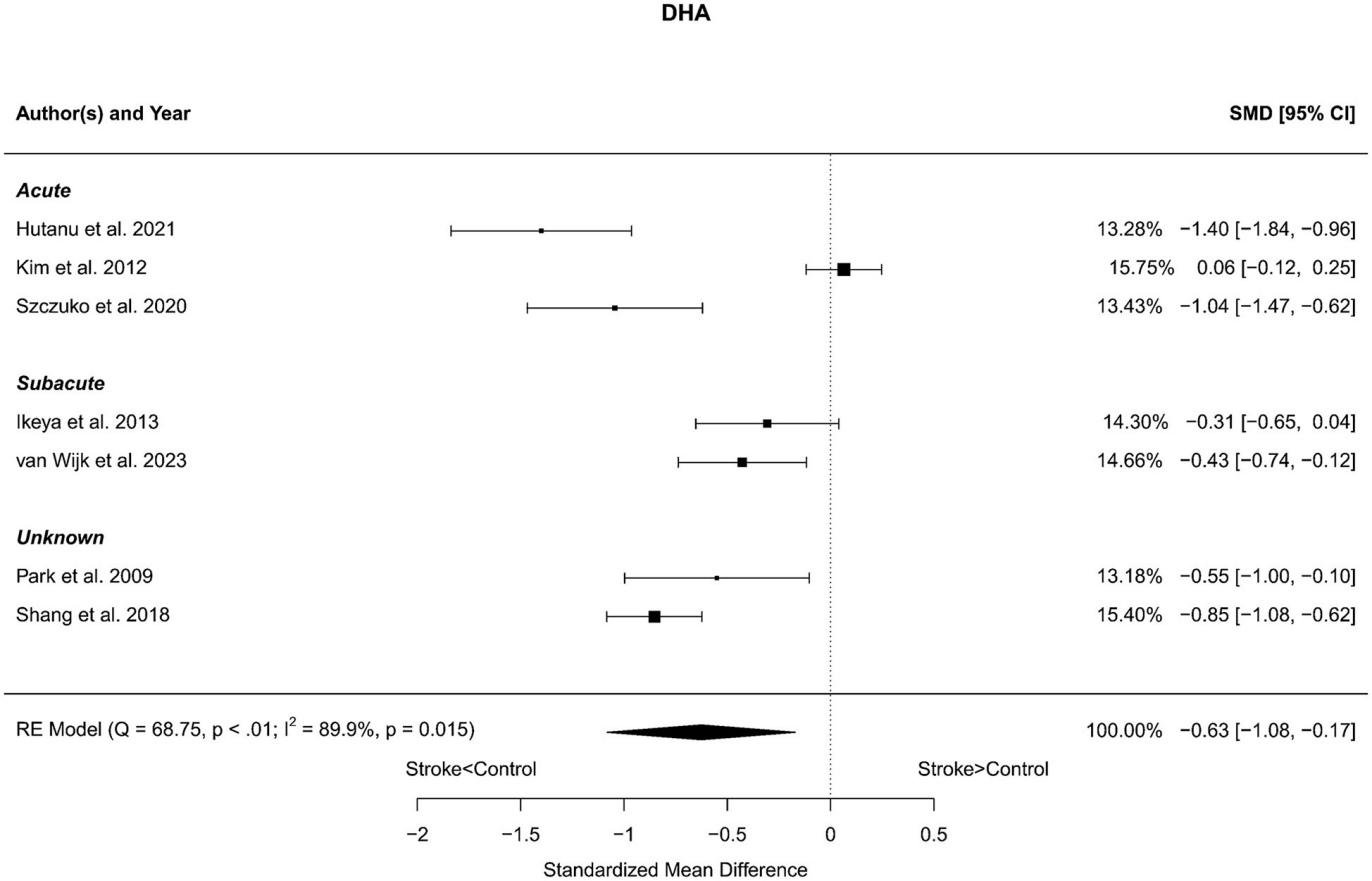
Comparison of blood DHA levels in stroke patients and controls. Included reports are organized in the forest plot by phase (acute—subacute—unknown) to visualize possible effects of time after stroke. Relative weights (%) and standardized mean differences (plus confidence interval) for each report are indicated at the right. Characteristics and result of the RE meta-analysis are in the lower left corner.

Seven reports with a total of 773 stroke cases and 608 controls were included in the meta-analysis (Park et al., 2009; Kim et al., 2012; Ikeya et al., 2013; Shang et al., 2018; Szczuko et al., 2020; Hutanu et al., 2021; van Wijk et al., 2023). The mean number of subjects was 110.4 (range: 40–248) in the stroke groups and 86.9 (range: 35–215) in the control groups. The mean age was 66.8 (range: 59.0–72.5) years for stroke cases and 65.3 (range: 54.4–75.4) years for controls. Blood levels of DHA were lower in stroke patients compared to controls for both the unadjusted [*t*_(6)_ = −3.37, *p* = 0.015] and age-adjusted [*t*_(5)_ = −3.66, *p* = 0.015] model (Figure 6).

### 3.7 Eicosapentaenoic acid

**Figure 7:**
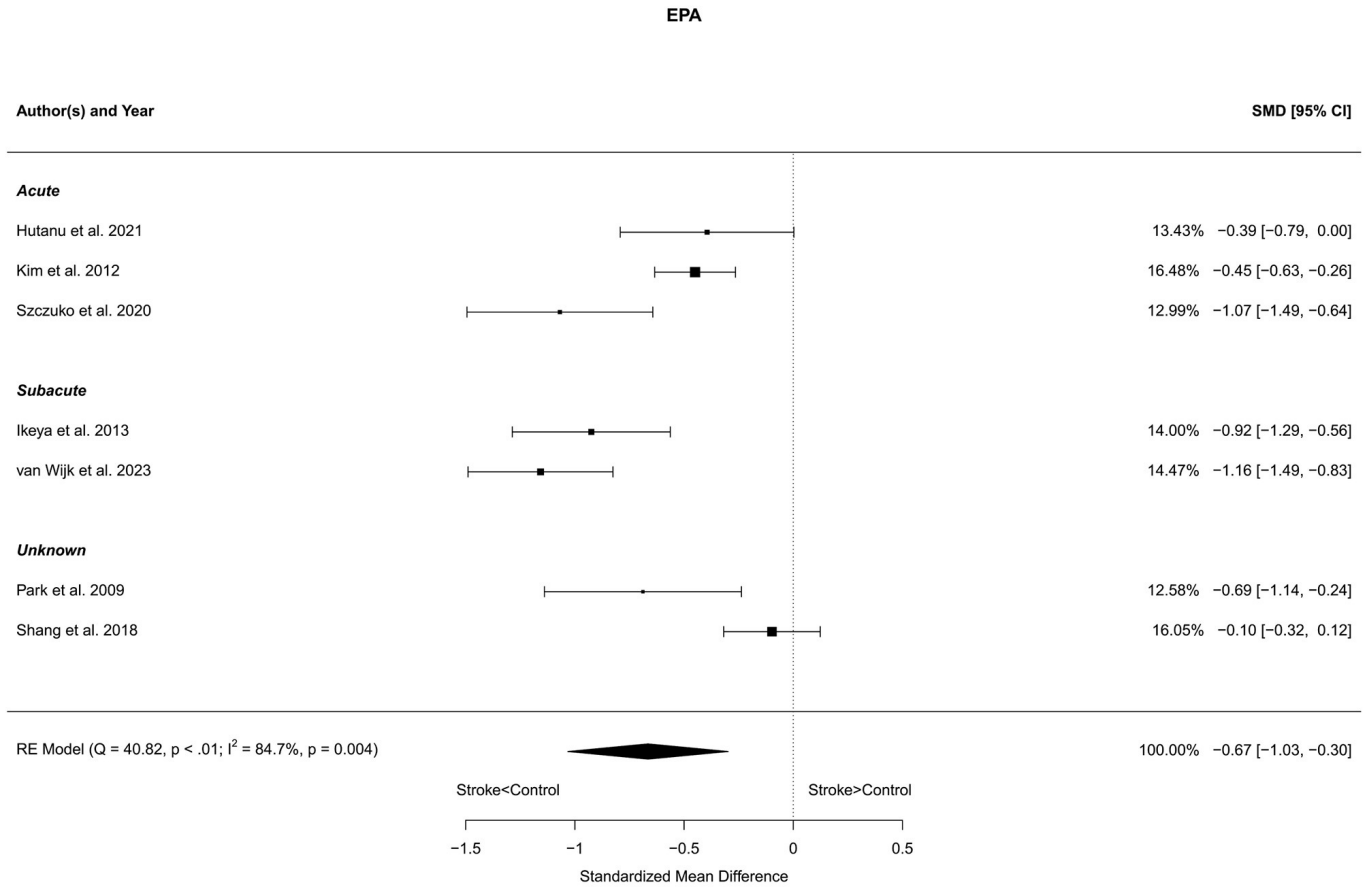
Comparison of blood EPA levels in stroke patients and controls. Included reports are organized in the forest plot by phase (acute—subacute—unknown) to visualize possible effects of time after stroke. Relative weights (%) and standardized mean differences (plus confidence interval) for each report are indicated at the right. Characteristics and result of the RE meta-analysis are in the lower left corner.

Seven reports with a total of 773 stroke cases and 608 controls were included in the meta-analysis (Park et al., 2009; Kim et al., 2012; Ikeya et al., 2013; Shang et al., 2018; Szczuko et al., 2020; Hutanu et al., 2021; van Wijk et al., 2023). The mean number of subjects was 110.4 (range: 40–248) in the stroke groups and 86.9 (range: 35–215) in the control groups. The mean age was 66.8 (range: 59.0–72.5) years for stroke cases and 65.3 (range: 54.4–75.4) years for controls. Blood levels of EPA were lower in stroke patients compared to controls for both the unadjusted [*t*_(6)_ = −4.44, *p* = 0.004] and age-adjusted [*t*_(5)_ = −4.20, *p* = 0.009] model (Figure 7).

### 3.8 Vitamin B6

**Figure 8:**
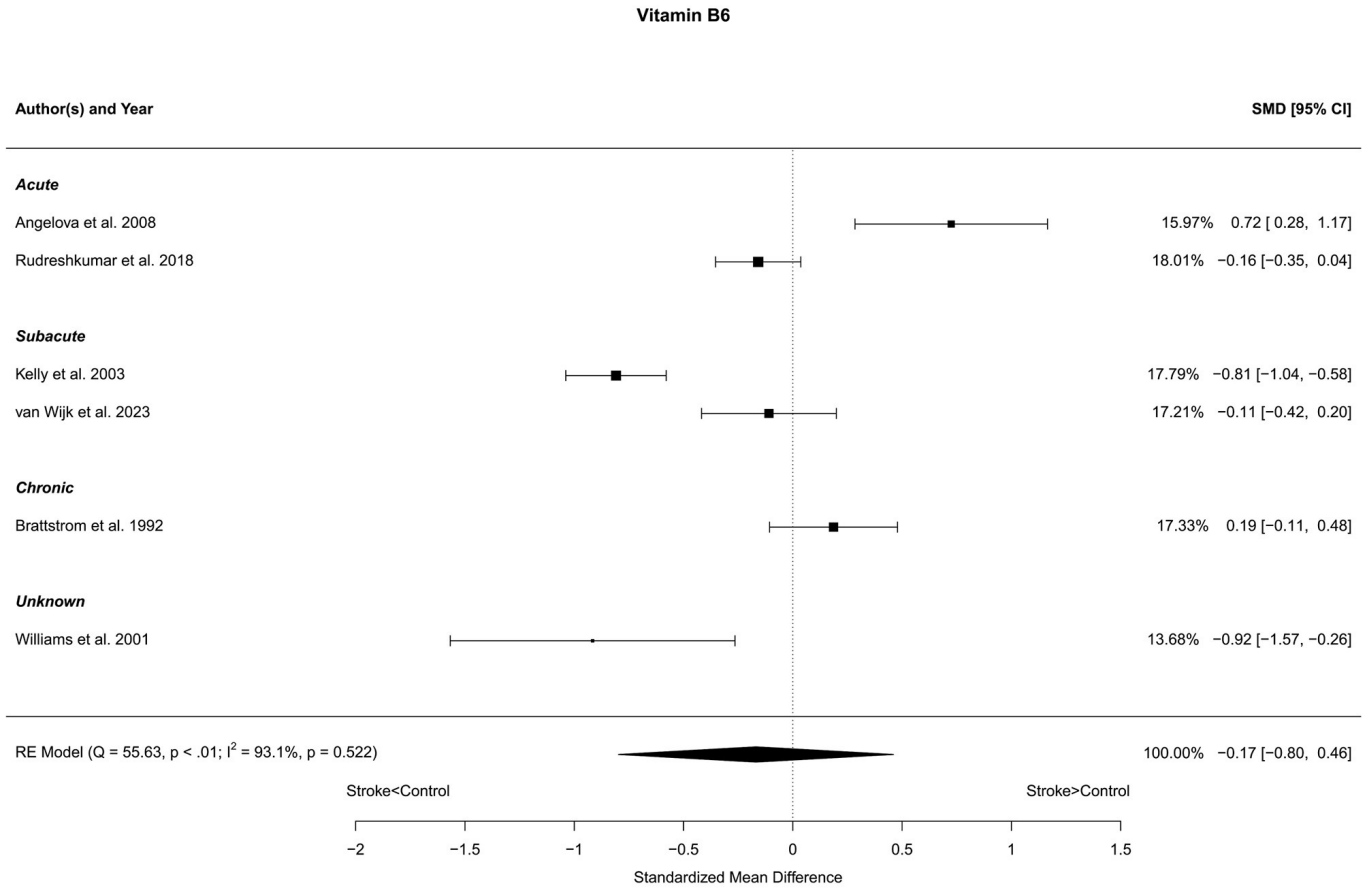
Comparison of blood vitamin B6 levels in stroke patients and controls. Included reports are organized in the forest plot by phase (acute—subacute—chronic—unknown) to visualize possible effects of time after stroke. Relative weights (%) and standardized mean differences (plus confidence interval) for each report are indicated at the right. Characteristics and result of the RE meta-analysis are in the lower left corner.

Six reports with a total of 631 stroke cases and 604 controls were included in the meta-analysis (Brattstrom et al., 1992; Williams et al., 2001; Kelly et al., 2003; Angelova et al., 2008; Rudreshkumar et al., 2018; van Wijk et al., 2023). The mean number of subjects was 105.2 (range: 20–180) in the stroke groups and 100.7 (range: 20–249) in the control groups. The mean age was 56.1 (range: 27.7–70.7) years for stroke cases and 53.6 (range: 27.0–68.4) years for controls. Blood levels of vitamin B6 were not different in stroke patients compared to controls; neither for the unadjusted [*t*_(5)_ = −0.69, *p* = 0.52], nor for age-adjusted [*t*_(4)_ = −0.62, *p* = 0.57] model (Figure 8).

### 3.9 Vitamin C

**Figure 9:**
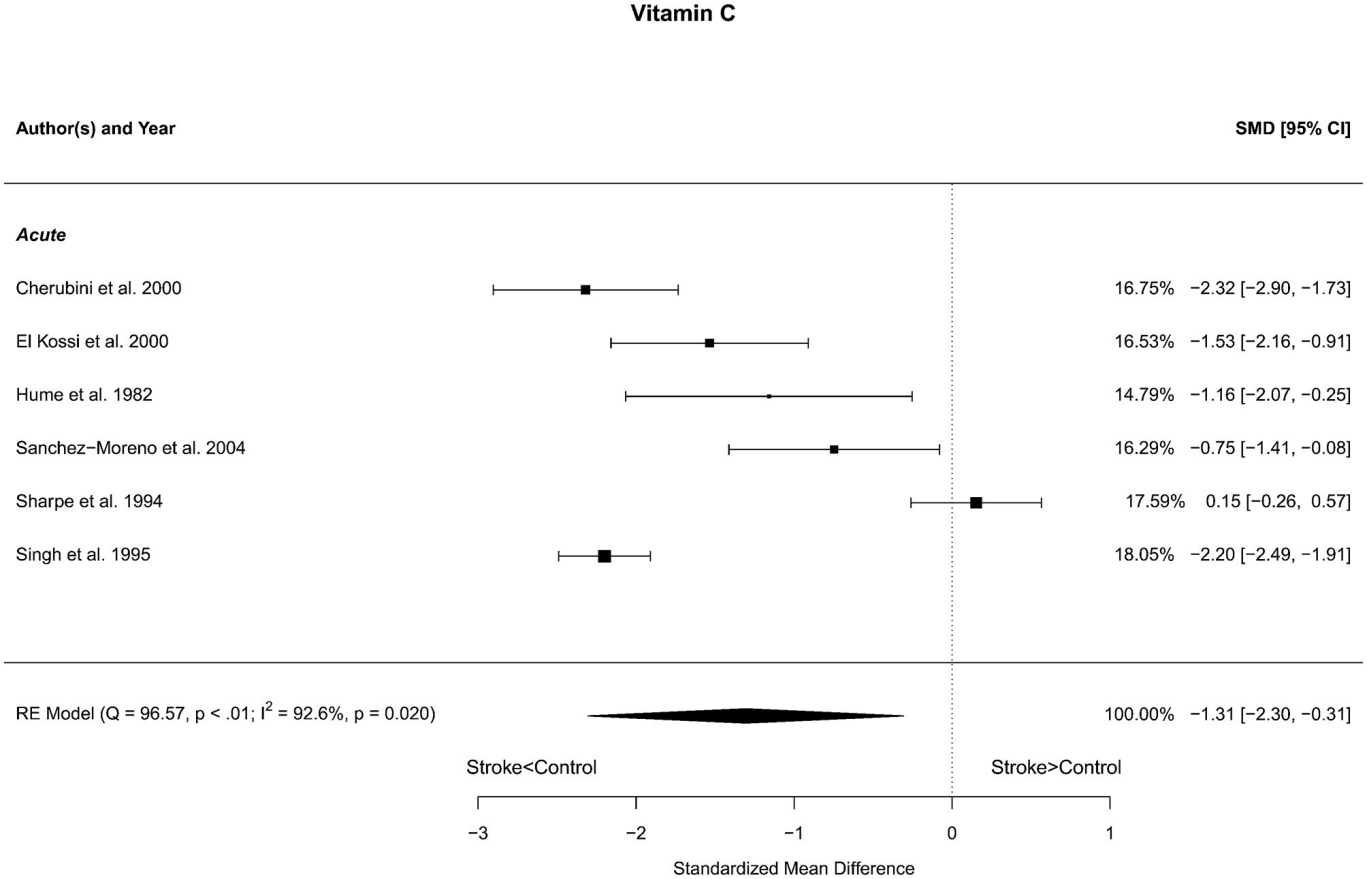
Comparison of blood vitamin C levels in stroke patients and controls. Included reports are all from the acute phase after stroke. Relative weights (%) and standardized mean differences (plus confidence interval) for each report are indicated at the right. Characteristics and result of the RE meta-analysis are in the lower left corner.

Six reports with a total of 248 stroke cases and 342 controls were included in the meta-analysis (Hume et al., 1982; Sharpe et al., 1994; Singh et al., 1995; Cherubini et al., 2000; El Kossi and Zakhary, 2000; Sanchez-Moreno et al., 2004). The mean number of subjects was 41.3 (range: 10–110) in the stroke groups and 57.0 (range: 12–202) in the control groups. The mean age was 65.0 (range: 52.5–77.2) years for stroke cases and 62.0 (range: 46.2–77.8) years for controls. Blood levels of vitamin C were significantly lower in stroke patients compared to controls, with statistical significance for the unadjusted [*t*_(5)_ = −3.36, *p* = 0.020] and a trend in the age-adjusted [*t*_(3)_ = −2.49, *p* = 0.088] model (Figure 9).

### 3.10 Selenium

**Figure 10:**
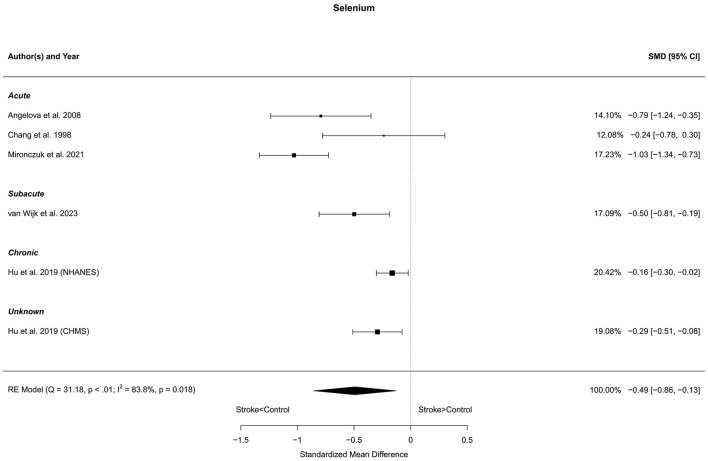
Comparison of blood selenium levels in stroke patients and controls. Included reports are organized in the forest plot by phase (acute—subacute—chronic—unknown) to visualize possible effects of time after stroke. Relative weights (%) and standardized mean differences (plus confidence interval) for each report are indicated at the right. Characteristics and result of the RE meta-analysis are in the lower left corner.

Six reports with a total of 581 stroke cases and 12,030 controls were included in the meta-analysis (Chang et al., 1998; Angelova et al., 2008; Hu et al., 2019; Mironczuk et al., 2021; van Wijk et al., 2023). The mean number of subjects was 96.8 (range: 36–202) in the stroke groups and 2,005 (range: 21–6,983) in the control groups. The mean age was 63.0 (range: 46.9–70.8) years for stroke cases and 52.9 (range: 46.0–63.1) years for controls. Blood levels of selenium were lower in stroke patients compared to controls, for both unadjusted [*t*_(5)_ = −3.47, *p* = 0.018] and age-adjusted [*t*_(4)_ = −3.19, *p* = 0.033] model (Figure 10).

### 3.11 Arginine

**Figure 11:**
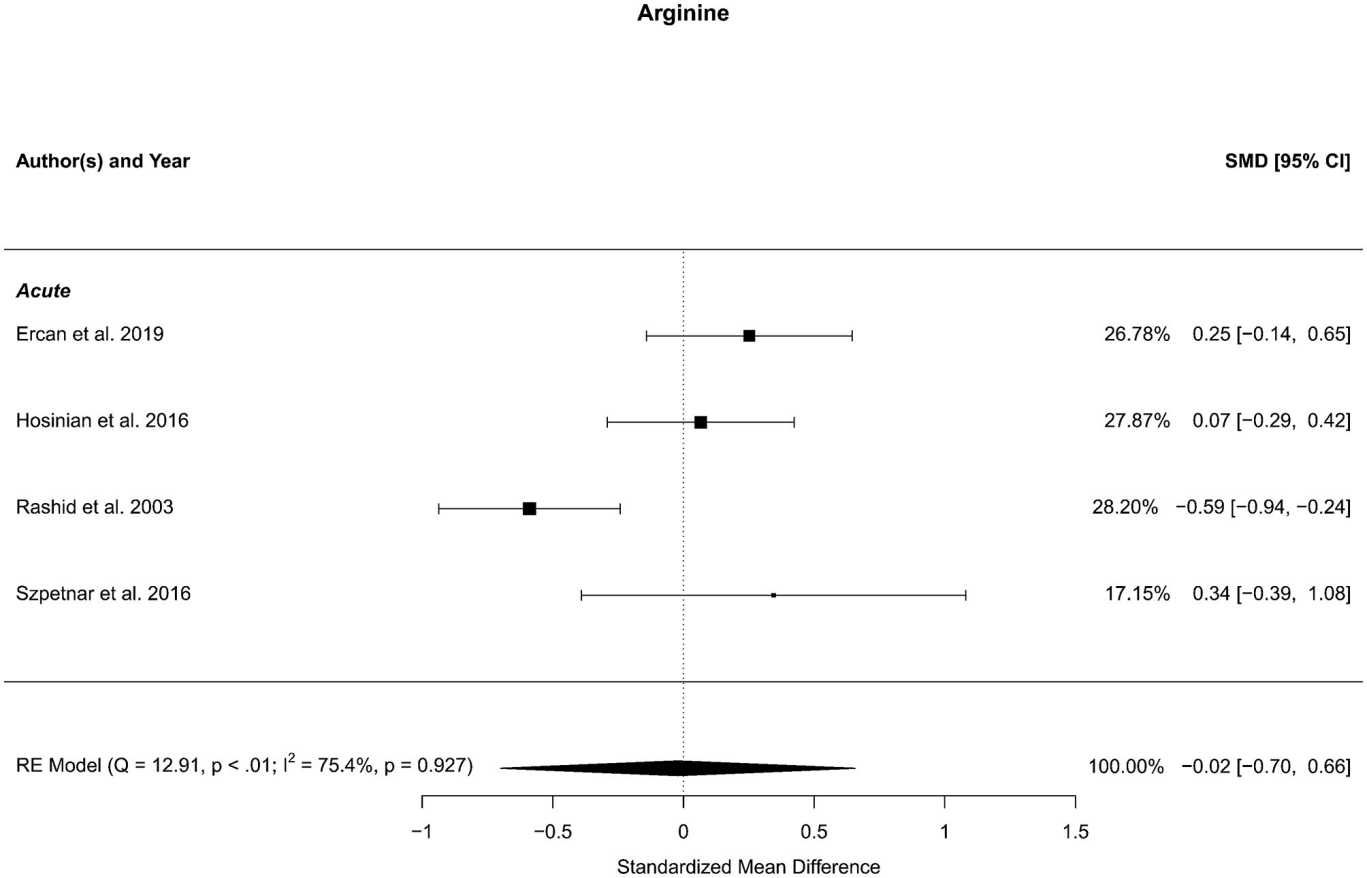
Comparison of blood arginine levels in stroke patients and controls. Included reports are all from the acute phase after stroke. Relative weights (%) and standardized mean differences (plus confidence interval) for each report are indicated at the right. Characteristics and result of the RE meta-analysis are in the lower left corner.

Four reports with a total of 358 stroke cases and 158 controls were included in the meta-analysis (Rashid et al., 2003; Hosinian et al., 2016; Szpetnar et al., 2016; Ercan et al., 2019). The mean number of subjects was 89.5 (range: 18–228) in the stroke groups and 39.5 (range: 12–60) in the control groups. The mean age was 71.0 (range: 64.0–75.3) years for stroke cases and 65.7 (range: 60.1–71.3) years for controls. Blood levels of arginine were not significantly different in stroke patients and controls; neither for unadjusted [*t*_(3)_ = −0.10, *p* = 0.93], nor for age-adjusted [*t*_(2)_ = −0.12, *p* = 0.92] model (Figure 11).

### 3.12 Not included in the meta-analysis: taurine, CoQ10, choline, carnitine, and uridine

For five of the preselected nutrients, i.e., taurine, CoQ10, choline, carnitine, and uridine, an insufficient number of reports was found to perform the meta-analysis.

For taurine, two reports with a total of 188 stroke cases and 97 controls were selected (Castillo et al., 1996; Ghandforoush-Sattari et al., 2011). The mean number of subjects was 94.0 (range: 60–128) in the stroke groups and 48.5 (range: 43–54) in the control groups. The mean reported age was 68.0 years for stroke cases and 56.0 years for controls. In both studies blood levels of taurine in stroke patients were elevated and reached statistical significance in one out of two (*p* < 0.0001 vs. *p* = 0.170) studies.

For CoQ10, two reports with a total of 157 stroke cases and 117 controls were selected (Simani et al., 2018; van Wijk et al., 2023). The mean number of subjects was 78.5 (range: 76–81) in the stroke groups and 58.5 (range: 34–83) in the control groups. The mean age was 63.6 (range: 63.0–64.2) years for stroke cases and 62.0 (range: 60.8–63.1) years for controls. Both studies reported lower blood levels of CoQ10 in stroke patients compared to controls (*p* < 0.05 and *p* < 0.001).

For choline, two reports with a total of 145 stroke cases and 148 controls were selected (Lee et al., 2019; van Wijk et al., 2023). The mean number of subjects was 72.5 (range: 65–80) in the stroke groups and 74.0 (range: 65–83) in the control groups. The mean age was 63.7 (range: 63.2–64.2) years for stroke cases and 62.2 (range: 61.3–63.1) years for controls. Both studies reported lower blood levels of choline in stroke compared to controls (*p* < 0.002 and *p* < 0.008).

Only one report (van Wijk et al., 2023) showed that blood levels of uridine was 4.01 ± 1.28 μmol/L in 78 stroke cases and 4.17 ± 1.24 μmol/L in 82 controls (*p* > 0.1). The blood levels of total carnitine reported in the same study for 81 stroke cases and 82 controls were 49.2 ±13.6 and 51.3 ± 9.4 μmol/L (*p* > 0.1), respectively.

## 4 Discussion

The main findings of our systematic review are that stroke patients have lower blood levels of many nutrients that are involved in repair processes after stroke, including folate, vitamin B12, EPA, DHA, vitamin C, vitamin E, selenium, and possibly CoQ10 and choline. These low blood levels highlight the presence of a suboptimal nutritional status after stroke. Our findings indicate that the inclusion of targeted nutritional interventions to further support recovery should receive consideration in the multidisciplinary context of stroke rehabilitation.

This systematic review shows that nutritional insufficiencies are quite common in stroke patients. From a selected group of nutrients relevant for their involvement in the response to injury and repair processes in the central nervous system, nine nutrients yielded a sufficient number of reports to be included in the meta-analysis. For seven out of these nine nutrients, the blood levels were found to be significantly lower in stroke patients compared to controls. These nutrients comprised B-vitamins (vitamin B12, folate), antioxidants (vitamin C, vitamin E, selenium), and long chain n-3 polyunsaturated fatty acids (n-3 PUFAs: DHA, EPA). In addition, there were five nutrients with an insufficient number of reports to perform the meta-analysis. For two of these nutrients, choline and CoQ10, studies predominantly reported lower blood levels in stroke patients compared to controls. For the nutrient taurine, studies reported higher blood levels in stroke patients compared to controls. Overall, the risk of bias in the selected studies was low, due to the non-randomized observational nature of the studies and the use of objective analytical assessments. If the present bias had affected the result of the meta-analysis, it is expected to have resulted in a slight moderation of the observed insufficiencies.

The majority of reports included in the present meta-analysis studied blood nutrient levels in the acute phase, i.e., within the first week after the stroke. However, we observed no clear differences between findings from the acute, subacute or chronic phases; in every phase post-stroke, lower levels are observed. Furthermore, individual findings that seemed to deviate from the observed trends could not be linked to a specific period of measurement. It is important to realize that individual nutrients may differ considerably in kinetic properties related to their uptake, storage, and secretion. Nevertheless, even blood levels of nutrients with larger storage capacities, like vitamin B12 and the n3-PUFAs EPA and DHA, were found to be low in stroke patients. Thus, the present observations suggest that specific nutrient insufficiencies are already present at the time of stroke and/or quickly develop after stroke.

The observed number of nutritional insufficiencies after stroke raises the question whether the present findings merely reflect the high prevalence of malnutrition, a reduced dietary intake, and the presence of dysphagia that is commonly observed after stroke. Indeed, a recent systematic review and meta-analysis showed that an impaired nutritional condition may already be present at the time of a stroke, but is also known to develop or to deteriorate in the months after stroke (Huppertz et al., 2021). Unfortunately, reports included in the present analysis have, in general, not provided specific information on the prevalence of dysphagia and/or malnutrition in their patient populations. It is clear that there is a need for longitudinal studies to provide more detail on the development of specific nutritional insufficiencies over time and their link to protein-energy malnutrition. Still, nutritional insufficiencies after stroke are also observed in non-dysphagic patients and even in well-nourished patients without overt signs of malnutrition (van Wijk et al., 2023), indicating that additional factors contribute to the weakened nutritional status of stroke patients. Stroke is an acute condition that may have long-term consequences; it triggers a cascade of physiological responses and, depending on the site and the size of the infarcted area, may result in long-term metabolic changes related to inactivity-related muscle wasting, wound healing and chronic inflammation (Wieloch and Nikolich, 2006; Aquilani et al., 2011; Dalise et al., 2014; Hajsl et al., 2020).

Because nutritional insufficiencies can already be observed in acute stroke patients, they could have been present before the stroke and could be the result of an interplay of pre-stroke lifestyle factors, associated comorbidities, and the use of related medication. It is well-known that lifestyle factors including diet and aerobic exercise contribute to important vascular and metabolic risk factors for stroke (Suter, 1999; Karttunen et al., 2002; He et al., 2004; Myint et al., 2008; Brouwer et al., 2021). Similarly, such lifestyle factors contribute to the presence and severity of several common comorbidities in stroke patients, including hyperhomocysteinemia, type 2 diabetes mellitus, high blood pressure, and high plasma lipids. As a consequence, polypharmacy is not uncommon in stroke patients, and the regular use of certain medication is known to influence the intake, metabolism, and excretion of nutrients (Gervasio, 2010; Wakeman and Archer, 2020). Deficiencies in the affected nutrients, e.g., vitamin B12, are likely to contribute to an exacerbation of problems, such as diabetic neuropathy (Wakeman and Archer, 2020). It therefore appears that stroke patients display nutritional insufficiencies that not only increase the risk of stroke and stroke recurrence, but also decrease the likelihood of functional recovery after stroke. There is strong preclinical evidence obtained in the transient middle cerebral artery occlusion mouse model of stroke, showing that a therapeutic dietary supplementation containing eight of the identified nutrients, i.e., DHA, EPA, vitamin C, vitamin E, selenium, vitamin B12, folic acid, and choline, was able to restore brain structure and function after ischemic stroke (Wiesmann et al., 2017). In this study, dietary supplementation decreased neuroinflammation, improved functional and structural brain connectivity, improved cerebral blood flow in the affected area, and also improved motor function in the mice.

Given the importance of lifestyle factors in the causation of stroke, changes in lifestyle are likely to support the recovery process. Adherence to healthier diets, such as the Mediterranean diet, DASH diet, or MIND diet, that would be expected to increase the dietary intake of vegetables, fruits, and fatty fish, i.e., sources of B-vitamins, antioxidants, and n-3 PUFAs, could help to maintain the improved nutritional status after recovery. Since the implementation of and the adherence to dietary changes may take considerable time and effort, targeted nutritional products or dedicated food supplements could be considered to help addressing the specific nutritional needs.

To our knowledge, this systematic review has been performed in the most appropriate way to provide a transparent and comprehensive overview of the existing evidence. Nevertheless, this study has some limitations. The preselected list of nutrients that was used for the systematic review is certainly not exhaustive. It is therefore likely that additional nutritional insufficiencies will be discovered with further searches. Historically, some nutrients have been studied more often than others, due to their involvement in stroke-relevant processes or risk factors, and were therefore more likely to be included in this review. For future studies we recommend the inclusion of other nutrients that may be involved in recovery. In addition, we note that the included reports may have studied heterogenous populations and used somewhat different control groups, which may have affected the conclusions. Given the worldwide differences in lifestyle and dietary habits, it would be interesting to investigate the influence of geographical differences in the prevalence of specific nutritional insufficiencies. The relative low number of observations per country and nutrient did not allow further analysis in this direction. Similarly, the severity of the stroke and the associated complications may have an important impact on the nutritional status of stroke patients. Unfortunately, the included reports did not provide sufficient information to assess the potential influence of this factor. Despite these limitations, all eligible literature on blood levels of the selected nutrients in stroke was considered valuable and included in the analysis of the review. More research should be carried out to further clarify changes in nutrient levels over time after stroke. Nevertheless, our results shed light on a problem that is often underestimated or even unnoticed, but is likely to be most relevant for functional recovery after stroke.

The current findings may have important consequences for how we appreciate the value of adequate nutrition and the supplementation of those nutrients that can address the condition-specific nutritional needs of patients in recovery after stroke. Current international stroke guidelines do not recommend routine administration of generic oral nutritional supplements in well-nourished stroke patients (Burgos et al., 2018; Powers et al., 2018; NICE, 2019). Based on our findings, it can be argued that the assessment of nutritional status in stroke should not be limited to the detection of protein-energy malnutrition and that tailored nutritional supplementation should be considered as a possibility in order to support optimal functional recovery after stroke.

## Data availability statement

The original contributions presented in the study are included in the article/Supplementary material, further inquiries can be directed to the corresponding author.

## Author contributions

LB: Investigation, Visualization, Writing—original draft, Writing—review & editing, Validation. SG: Writing— original draft, Writing—review & editing, Investigation. AC-Y: Formal analysis, Methodology, Validation, Visualization, Writing—review & editing. NW: Investigation, Visualization, Writing—review & editing. AH: Conceptualization, Writing—review & editing. AM-T: Conceptualization, Writing— review & editing. ML: Conceptualization, Supervision, Writing—review & editing.
