# Supplementary material for: Stroke patients have lower blood levels of nutrients that are relevant for recovery: a systematic review and meta-analysis
Source: Front Stroke. 2023 Dec 13;2:1274555. doi: 10.3389/fstro.2023.1274555 (PMC12802784; doi:10.3389/fstro.2023.1274555)
Supplement: Supplementary file 1 [file Data_Sheet_1.docx]

Supplementary Material

# Supplementary Tables and Figures

## Supplementary Tables

**Table S1**: Search strategy.

| **Meta-analysis 1980-2022** | |
| --- | --- |
| Conceptual String: | Ti,ab((cerebrovascular) AND (ischemic) AND (nutrition) AND (selected nutrients) NOT (animal)) |
| String: | Ti,ab((((cerebrovascular OR cerebral OR brain) AND (haemorrhage OR hemorrhage OR accident OR lesion OR vasculopathy OR attack OR disturbance OR ischemic OR ischaemic OR ischemia OR ischaemia OR arrest OR failure OR seizure OR infarct OR infarction OR insult OR reperfusion)) OR CVA OR "cerebrovascular accident" OR TIA OR "transient ischemic attack" OR "transient ischaemic attack" OR CVI OR apoplexy OR apoplexi* OR stroke* OR poststroke OR “post stroke” OR "post-stroke") AND (“oral supplement*” OR “oral feed*” OR “oral nutrition” OR “oral nutritional supplement*” OR malnutrition OR malnourish* OR undernutrition OR undernourish* OR deficiency OR intake OR status OR deficient OR deficiencies OR nutrition OR nutrient* OR dietary OR blood OR plasma OR serum OR RBC OR "red blood cells" OR erythrocyte*) AND (DHA OR docosahexaenoic p/0 acid OR docosahexanoic p/0 acid OR "omega-3 fatty acid*" OR EPA OR eicosapentaenoic p/0 acid OR eicosapentaenoic p/0 acid OR UMP OR uridine OR uridine p/0 monophosphate OR "B-complex vitamin*" OR "B complex vitamin*" OR choline OR phospholipid* OR folic p/0 acid OR folate OR “vitamin b9” OR "vitamin M" OR "vitamin Bc" OR pteroyl-L-glutamate OR pteroyl-L-glutamic p/0 acid OR folacin OR “vitamin B6” OR pyridoxine OR pyridoxal OR pyridoxamine OR “vitamin b12” OR cobalamin OR cyanocobalamin OR “vitamin C” OR ascorbic p/0 acid OR ascorbate OR “vitamin E” OR tocopherol OR selenium OR carnitine OR taurine OR cysteine OR arginine OR “coenzyme q10” OR co-enzyme p/0 Q10 OR coenzyme p/0 Q-10 OR co-enzyme p/0 Q-10 OR coq10 OR ubiquinone OR ubidecarenone OR coq OR q10 OR benzoquinone OR co-enzyme p/0 Q OR “coenzyme q” OR “vitamin K” OR tocotrienol) NOT (animal OR mice OR mouse OR rats OR murine OR model OR dog* OR cat* OR canine OR rabbit* OR gerbil* OR “in vitro” OR “in vivo” OR poultry OR cattle)) |
| Databases: | Embase, MEDLINE |
| Publication date: | Published between 01-01-1980 and 01-10-2022 |
| Language: | English |
| Document Type Exclusions: | Conference Abstract AND Conference Paper AND Conference paper AND Short Survey AND Letter AND Book chapter AND Retracted Publication AND Comment AND Editorial AND Note AND Book AND Conference Review AND Duplicate Publication AND Thesis AND Tombstone AND Bulletin article AND Chapter AND Clinical Conference AND Congress AND Correspondence AND Erratum AND Journal issue AND News AND Video-Audio Media |
| # of results: | 5893 original Journal Articles |

**Table S2**: Summary of the results.

| **Nutrient** | **Reports  (k)** | **Overall result** |
| --- | --- | --- |
| Folate | 27 | ↓ |
| Vitamin B12 | 23 | ↓ |
| Vitamin E | 11 | ↓ |
| DHA | 7 | ↓ |
| EPA | 7 | ↓ |
| Vitamin B6 | 6 | ─ |
| Vitamin C | 6 | ↓ |
| Selenium | 6 | ↓ |
| Arginine | 4 | ─ |
| Taurine | 2 | (↑) |
| Choline | 2 | (↓) |
| CoQ10 | 2 | (↓) |
| Carnitine | 1 | (─) |
| Uridine | 1 | (─) |

## Supplementary Figures

**
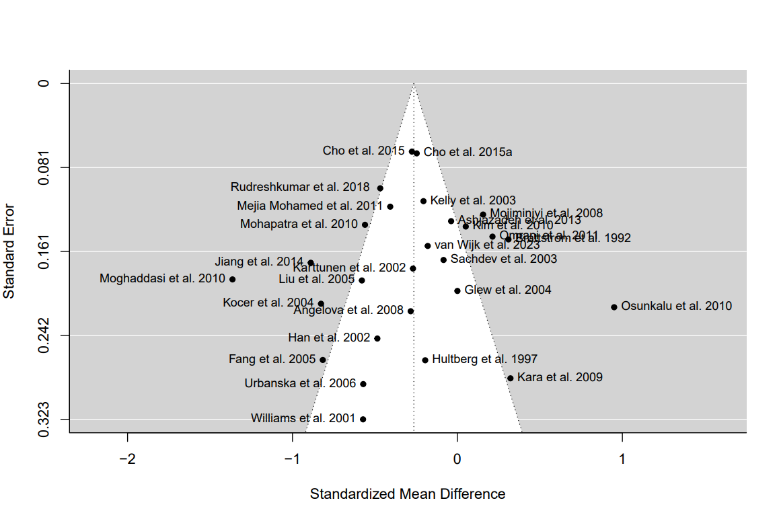
**

**Figure S1**: Funnel plot for reports on blood folate levels.

**
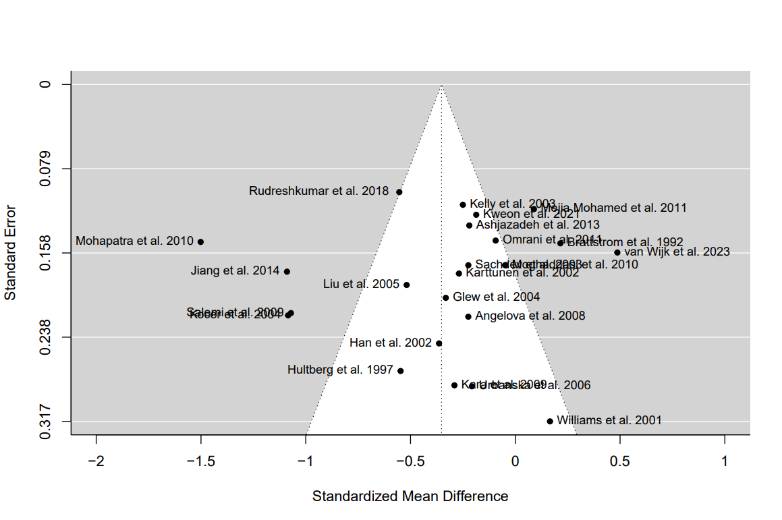
**

**Figure S2**: Funnel plot for reports on blood vitamin B12 levels.

**
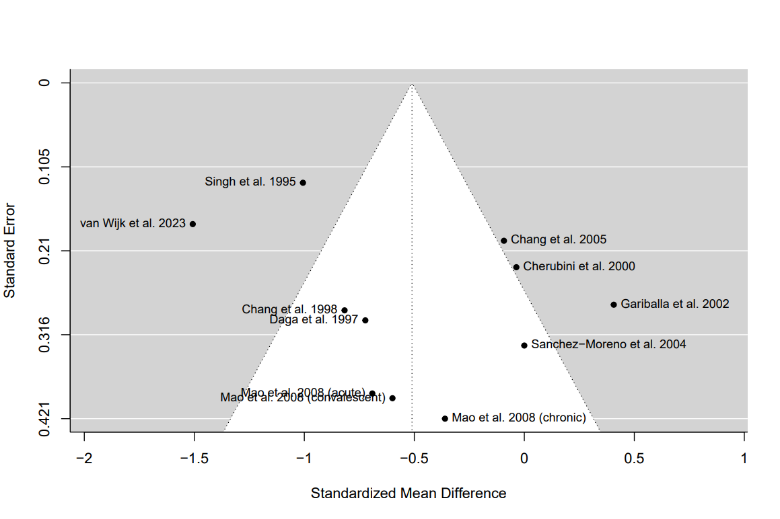
**

**Figure S3**: Funnel plot for reports on blood vitamin E levels.
